# An individualized tractography pipeline for the nucleus basalis of Meynert lateral tract

**DOI:** 10.1162/imag_a_00067

**Published:** 2024-01-11

**Authors:** Rachel A. Crockett, Kevin B. Wilkins, Michael M. Zeineh, Jennifer A. McNab, Jaimie M. Henderson, Vivek P. Buch, Helen M. Brontë-Stewart

**Affiliations:** Department of Neurology and Neurological Sciences, Stanford University School of Medicine, Stanford, CA, United States; Department of Radiology, Stanford University School of Medicine, Stanford, CA, United States; Wu Tsai Neurosciences Institute, Stanford University, Stanford, CA, United States; Bio-X, Stanford University, Stanford, CA, United States; Department of Neurosurgery, Stanford University School of Medicine, Stanford, CA, United States

**Keywords:** Tractography, cholinergic network, nucleus basalis of Meynert, neurosurgery, deep brain stimulation

## Abstract

*Background:* At the center of the cortical cholinergic network, the nucleus basalis of Meynert (NBM) is crucial for the cognitive domains most vulnerable in Parkinson’s disease (PD). Preclinical evidence has demonstrated the positive impact of NBM deep brain stimulation (DBS) on cognition but early human trials have had mixed results. It is possible that DBS of the lateral NBM efferent white matter fiber bundle may be more effective at improving cognitive-motor function. However, precise tractography modelling is required to identify the optimal target for neurosurgical planning. Individualized tractography approaches have been shown to be highly effective for accurately identifying DBS targets but have yet to be developed for the NBM.

*Methods:* Using structural and diffusion-weighted imaging, we developed a tractography pipeline using manually segmented regions of interest for precise individualized identification of the lateral NBM target tract. Using dice similarity coefficients, the reliability of the tractography outputs was assessed across three cohorts to investigate: 1) whether this manual segmentation pipeline is more reliable than an existing automatic segmentation pipeline currently used in the literature; 2) the inter- and intra-rater reliability of our pipeline in research scans of patients with PD; and 3) the reliability and practicality of this pipeline in clinical scans of DBS patients.

*Results:* The individualized manual pipeline was found to be significantly more reliable than the existing automated pipeline for both the segmentation of the NBM region itself (p < 0.001) and the reconstruction of the target lateral tract (p = 0.002). There was also no significant difference between the reliability of two different raters in the PD cohort (p = 0.25), which showed high inter- (mean Dice coefficient >0.6) and intra-rater (mean Dice coefficient >0.7) reliability across runs. Finally, the pipeline was shown to be highly reliable within the clinical scans (mean Dice coefficient = 0.77). However, accurate reconstruction was only evident in 7/10 tracts.

*Conclusion:* We have developed a reliable tractography pipeline for the identification and analysis of the NBM lateral tract in research and clinical-grade imaging of healthy young adult and PD patient scans.

## 1 Introduction

Disability and death due to Parkinson’s disease (PD) is increasing faster than any other neurological disorder (World Health Organization, 2022). While the cardinal motor symptoms of PD, bradykinesia, rigidity, and tremor (Sveinbjornsdottir, 2016) can be improved by dopaminergic interventions, treatments for cognitive impairment remain scarce and ineffective. The nucleus basalis of Meynert (NBM) is at the center of the cortical cholinergic network and is crucial for the cognitive domains most vulnerable in PD (A. K. L. Liu et al., 2015). Preclinical evidence has demonstrated the positive impact of NBM deep brain stimulation (DBS) on cognition (R. Liu et al., 2017, 2018) but early evidence in humans has had mixed results (Cappon et al., 2022; Freund et al., 2009; Gratwicke et al., 2020; Hardenacke et al., 2016; Kuhn et al., 2015). It is possible that the lateral NBM efferent white matter fiber bundle may be a novel and more effective DBS target for treating cognitive-motor syndrome. However, precise tractography modelling is required to identify the optimal target for neurosurgical planning.

The NBM is involved in tasks requiring attention, visuospatial, and executive functions (A. K. L. Liu et al., 2015). It is known to degenerate early in PD, which was predictive of cognitive but not motor impairment up to five years later (Ray et al., 2018). While reduced integrity of the NBM region has been shown to predict cognitive impairment in patients with PD (Schulz et al., 2018), reduced integrity of the NBM white matter fiber bundles was shown to have a stronger contribution to cognitive function than the NBM region itself (Q. Liu et al., 2017; Nemy et al., 2020; Schumacher et al., 2022). There are two main pathways emanating from the NBM: 1) a lateral pathway that travels through the external capsule and uncinate fasciculus to supply the frontal, insula, parietal, and temporal cortices; and 2) a medial pathway, passing through the cingulum to supply the parolfactory, cingulate, pericingulate, and retrosplenial cortices (Selden, 1998). Our previous work has shown greater mean diffusivity and reduced fractional anisotropy of both the lateral and medial NBM tracts in patients with early stage PD up to one year prior to the development of mild cognitive impairment (Crockett et al., 2023). However, only the lateral tract was associated with cognitive outcomes in older adults with mild cognitive impairment and dementia due to Alzheimer’s disease or dementia with Lewy bodies (Schumacher et al., 2022).

To date, DBS of the NBM in humans with Alzheimer’s disease (Hardenacke et al., 2016; Kuhn et al., 2015), dementia with Lewy bodies (Gratwicke et al., 2020), and PD (Cappon et al., 2022; Freund et al., 2009) have primarily targeted the grey matter nucleus itself with varying levels of symptom improvement. Evidence from the clinical depression literature indicates that targeting specific white matter fiber bundles may be more effective than exclusively focusing on grey matter nuclei targets. While targeting grey matter targets has been shown to be beneficial in ~60% of patients with depression (Figee et al., 2022), Riva-Posse et al. (2018) demonstrated that using individualized tractography to guide neurosurgical planning of the target of interest resulted in 82% of patients responding to DBS with over 50% in remission after 12 months. Similarly, tractography-guided DBS of the ventral intermediate nucleus of the thalamus was also found to be more successful at treating essential tremor, than targeting the nucleus directly (Fenoy & Schiess, 2018). Thus, tractography-guided approaches for DBS may have broad benefit.

The lateral NBM tract is a new target for DBS therapy in Parkinson’s disease (NCT05968703) and requires tractography-guided neurosurgical planning. Current methods for recreating the lateral NBM tract use automated segmentation of regions of interest (Nemy et al., 2020; Schumacher et al., 2022). Both of the most commonly used NBM masks from healthy adults were segmented by a probabilistic mask determined from magnetic resonance imaging and histology from between 1 to 10 post-mortem brains (Kilimann et al., 2014; Zaborszky et al., 2008). However, the reliability of using masks developed in post-mortem histology slices to in-vivo neuroimaging has been questioned (Wang et al., 2022). This is particularly challenging for small subcortical brain regions without very high scan resolution or stark contrast differences. In addition, these masks are developed from very small samples, which may not account for inter-subject variability, especially in clinical populations with a high level of atrophy. Thus, there is a need for more reliable individualized approaches to identify the NBM region and tract reconstruction. George et al. (2011) have developed a manual approach for segmenting the substantia innominata (SI) region, which contains the NBM, using structural neuroimaging. However, their approach was specific to volumetric analysis of the SI and did not separate the NBM from the surrounding anatomy. A manually segmented tractography approach for reconstructing the lateral NBM tract has yet to be evaluated.

Therefore, the aim of this study is to expand on these methods by using multi-modal neuroimaging and an individualized manual segmentation approach to develop an improved tractography methodology for the reconstruction of the NBM lateral tract. We aim to assess the reliability of our approach in three ways. First, we compare our manual segmentation pipeline to that of the existing automated pipeline used in the literature (Nemy et al., 2020) in a test-retest sample of high-quality research scans from healthy young adults. Second, given the manual nature of our proposed approach, we aim to confirm the reproducibility of its use across researchers by assessing the inter- and intra-rater reliability in research quality scans from a cohort of PD patients. Finally, to investigate the use of this pipeline in shorter clinical DTI scans, we will determine the test-retest reliability in a cohort of DBS PD patients from our database. The development of this pipeline will be beneficial both for more reliable investigation of the cortical cholinergic network as well as to guide the neurosurgical planning for DBS procedures that aim to target this region.

## 2 Methods

### 2.1 Participants

Cohort 1: Thirty-seven participants from the test-retest cohort from the Human Connectome Project (HCP) (Van Essen et al., 2012) were included in the first stage of our analyses. These participants were scanned on two separate occasions. Participants were included in the HCP study if they were: 1) aged 22-35 years old; 2) had no significant history of psychiatric disorder, substance abuse, neurological or cardiovascular disease; 3) had no evidence of cognitive impairment as indicated by a score ≥29 on the Mini Mental State Examination (MMSE); and 4) were able to provide informed consent. They were excluded if they: 1) had a history of seizures or epilepsy; 2) had a genetic disorder such as cystic fibrosis; 3) had been taking prescription medications for migraines in the last 12 months; 4) had multiple sclerosis, cerebral palsy, brain tumor, stroke, sickle cell disease, or experienced a severe head injury; 5) were currently on chemotherapy or immunomodulatory agent or had a history of this treatment that could affect the brain; 6) were currently being treated for diabetes; 7) were a premature birth; or 8) did not meet the safety criteria to undergo neuroimaging.

Cohort 2: Twenty-one participants with PD from the Parkinson’s Progression Markers Initiative (PPMI) database (www.ppmi-info.org/access-data-specimens/download-data, RRID:SCR_006431) were included in the cross-sectional second stage of this study (i.e., one scan). For up-to-date information on the PPMI study, visit www.ppmi-info.org. Participants were included in the PPMI study if they: 1) were ≥30 years old; 2) had PD for at least 2 years prior to screening; 3) were not currently on or expected to start PD medication for at least 6 months after baseline testing; 4) were not currently or planning to become pregnant during the study; and 5) were able to provide informed consent. They were excluded if they: 1) were currently taking PD medication; 2) had other atypical PD syndromes (e.g., metabolic disorders, encephalitis, or other neurodegenerative disease); 3) were clinically diagnosed with dementia; or 4) had any other medical or psychiatric condition which might preclude participation. Additional inclusion criteria for the current study required participants to have: 1) undergone magnetic resonance imaging (MRI) with both a structural T1-weighted (T1-w) and diffusion-weighted (DWI) scan; and 2) have evidence of mild cognitive impairment at baseline indicated by a score of 23-25 on the Montreal Cognitive Assessment (MoCA), which is consistent with the criteria for a patient to be eligible for NBM DBS to treat cognitive impairment.

Cohort 3: Five patients (aged 64-74 years, 1 female) with PD from our ongoing research investigating DBS of the subthalamic nucleus were included in the final cross-sectional clinical cohort (i.e., one scan). Participants were included in the parent study if they: 1) were over 18 years of age; 2) had a diagnosis of idiopathic PD; 3) had documented improvement in motor symptoms with dopaminergic medication; and 4) were on stable doses of medication. They were excluded if they: 1) were over 80 years of age; 2) had significant cognitive impairment or dementia as determined by a standardized neuropsychological battery; 3) were clinical depressed as defined by the DSM-IV; 4) had very advanced stage PD as indicated by Hoehn and Yahr stage 5 while on medication; 5) did not meet the requirements for MRI and/or neurosurgery; 6) had a history of seizures, or who required electroconvulsive therapy or transcranial magnetic stimulation to treat a chronic condition; 7) were unable to comply with study follow-up visits; or 8) were unable to provide informed consent.

### 2.2 Magnetic resonance imaging (MRI) acquisition

Cohort 1: Data from the HCP were acquired using a Siemens 3T scanner with high-performance head-only gradients. The T1-w images consisted of 256 slices, voxel size = 0.7 mm^3^, field of view = 224 x 224, repetition time (TR) = 2400 ms, and echo time (TE) = 2.14 ms. The diffusion tensor echo planar images (DTI) consisted of 111 slices with a right-to-left and left-to-right phase encoding direction, voxel size = 1.25 mm^3^, acquisition matrix = 168 x 144, TR = 5520 ms, TE = 89.5 ms, gradient directions = 90 (90 volumes for each shell) with *b= *1000, 2000, and 3000 s/mm^2^ and six *b*0 (*b* = 0 s/mm^2^) volumes interspersed throughout each run.

Cohort 2: The MRI scans from the PPMI database were acquired using standardized procedures across sites on a Siemens 3T scanner. The T1-w images consisted of 192 slices, voxel size = 1 mm^3^, acquisition matrix = 256 x 256, TR = 2300 ms, and TE = 2.98 ms. The DTI consisted of ~80 slices with a posterior-to-anterior phase encoding direction, voxel size = 2 mm^3^. The acquisition matrix = 128 x 128, TR = ~10000 ms, TE = ~80 ms, gradient directions = 64 with *b* = 1000 s/mm^2^ and one *b*0 image as the first volume, and acquisition time = ~95.5 s.

Cohort 3: The clinical MRI scans were acquired on a Discovery MR750 3T scanner at the Stanford Neuroscience Health Center. The T1-w image consisted of 188 slices, voxel size = 1 mm^3^, acquisition matrix = 256 x 256, TR = ~8.51 ms, and TE = ~3.2 ms. The DTI consisted of one 31 slice scan encoded with 30 gradient directions in the posterior to anterior direction and another consisting of two volumes with one gradient in the anterior to posterior direction. For both encoding directions, the voxel size = 2 mm^3^, acquisition matrix = 128 x 128, TR = ~8000 ms, TE = 61 ms, *b* = 1000 s/mm^2^ with one *b*0 image as the first volume of each run, and acquisition time = 90.6 s.

### 2.3 Tractography pipeline

Preprocessing of the MRI data was completed using FMRIB Software Library (FSL) (Jenkinson et al., 2012). Prior to completing the clinical tractography pipeline, topup (Andersson et al., 2003) and eddy (Andersson & Sotiropoulos, 2016) were implemented. Topup is used to correct for distortions caused by magnetic inhomogeneities along the phase encoding axis, while eddy is used to correct for eddy current induced distortions and subject movement. The data in cohort 1 had already undergone these preprocessing steps (Glasser et al., 2013). A diffusion tensor model was fit using FSL DTIFIT, producing three eigenvector images, mean diffusivity, and fractional anisotropy (FA) images. Removal of non-brain structures was completed using HD-BET (Isensee et al., 2019) on the T1-w, DTI and the *b*0 image. All images were then aligned to the anterior commissure-posterior commissure (ACPC) line using the rigid linear conversion from T1-w to the MNI ACPC line with six degrees of freedom as the reference transformation (Hayashi et al., 2018). The gradient direction matrices (bvecs) were also rotated to account for this transformation.

For both pipelines, the brainstem was extracted using FSL’s First automated segmentation tool (Patenaude et al., 2011), and the hemisphere mask was hand drawn in FSLeyes by identifying the midline of the brain in the coronal plane on the subject scans.

### 2.4 Manual segmentation of regions of interest

The optic tract and anterior commissure were identified using both the T1-w and FA maps and manually segmented using FSLeyes. The anterior end of the anterior commissure mask was identified by the slice at which there was a clear band across the coronal plane and was segmented through its full posterior extent. Both the T1-w and FA maps were used depending on the scan type that provided the clearest outline of the anatomy. In some instances, both scan types may be used for reference. This is consistent with guidelines for manual segmentation protocols using diffusion images (Rheault et al., 2022).

**Fig. 1.**
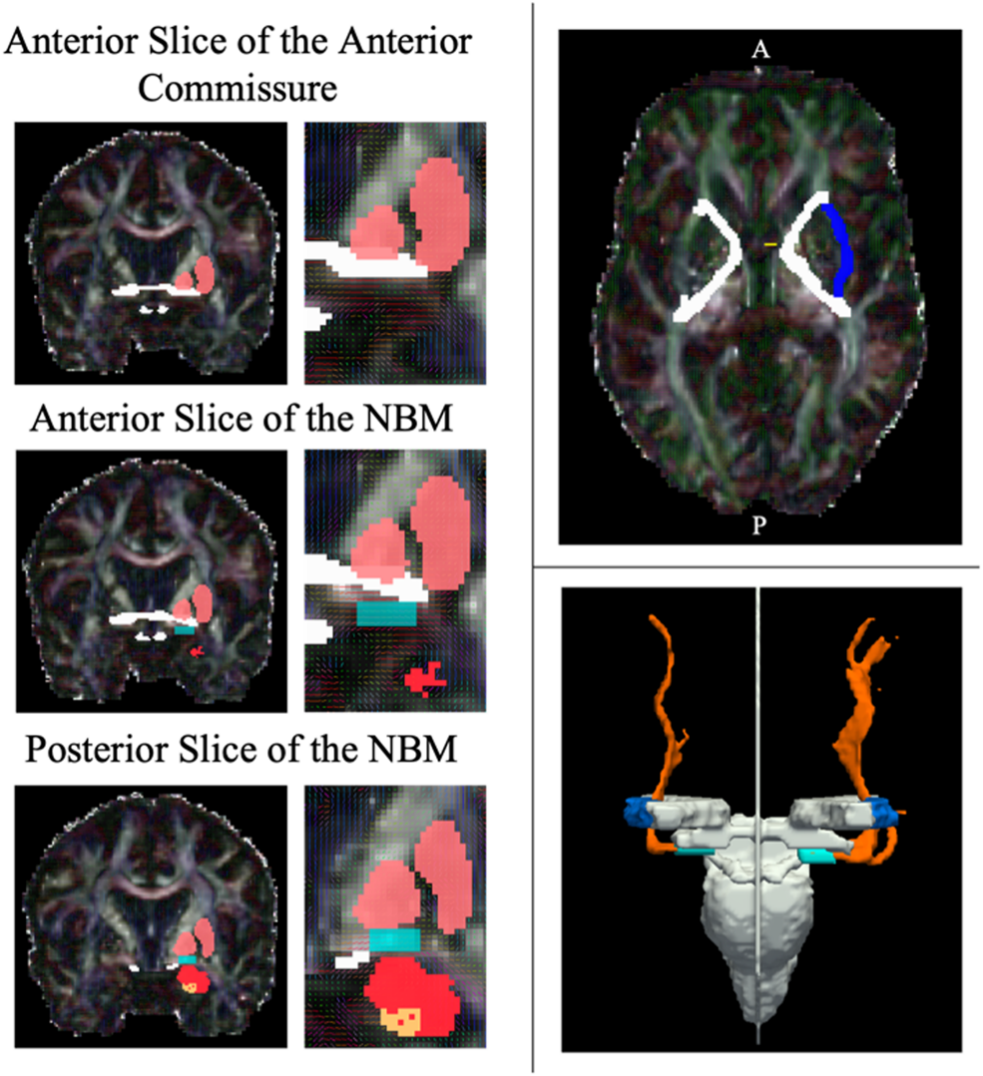
Left: Examples of the anterior and posterior slices of the NBM mask (cyan) using the pallidum/putamen (pink), anterior commissure and optic tract (white), amygdala (red), and hippocampus (orange) as guidance regions. Top Right: Example mask of the inferior slice of the internal capsule (white) not overlapping with the anterior commissure (yellow) and encapsulating the left external mask (navy). Bottom Right: Example tractography output (orange) with the seed NBM (cyan), external capsule (navy), and exclusion regions (white).

The external capsule was identified in the axial plane of the DWI (see Fig. 1). The most inferior slice began where there is a clear outline of the internal capsule that is not overlapping with the anterior commissure. From this slice, the external capsule is drawn stopping slightly short of the anterior and posterior ends of the capsule. To avoid the tractography being guided by overlapping tracts in the more superior portions of the external capsule, the superior boundary was specified at ~9 mm (7 slices for scans with a 1.25 mm voxel size) above the inferior slice.

The internal capsule mask is then identified across the same axial slices, ensuring that the anterior and posterior ends of the external capsules are sealed off by the internal capsule mask.

Finally, the NBM masks were identified in the coronal plane using a combination of the manually drawn anterior commissure and optic tract, and the automated extraction of the pallidum and hippocampus from the FSL First segmentation. Overlaying the outputted primary eigenvector image onto the FA image, the direction of diffusion in x, y, z is denoted in RGB colours whereby green = anterior-posterior, red = medial-lateral, and blue = superior-inferior. The NBM was identified by a strong lateral (red) directionality of the voxels below the pallidum and within the guidance anatomy (see Fig. 1). The most anterior slice of the anterior commissure (the point at which there is a strong and clear band across the coronal slice) was used to identify the most anterior portion of the NBM. Starting at least one slice posterior to this marker signified the first slice of the anterior NBM. In scans where identification of the anterior commissure was challenging, the point at which the inferior end of the fornix column meets the base of the internal capsule was used as an additional indicator of the anterior NBM boundary. The posterior slice was indicated at the point whereby the hippocampus begins to become evident. The medio-lateral border of the NBM was aligned with the medial and lateral edges of the pallidum. The anterior commissure and optic tract masks were used to avoid overlapping regions of the NBM. A slice-by-slice guide on the manual segmentation of the NBM in MNI space and using the slice numbers from the Atlas of the Human Brain (Mai et al., 2016) is provided in the Supplementary Material (S1).

### 2.5 Automated segmentation of regions of interest

The automated tractography pipeline was designed based on methods previously used for identifying the NBM tracts of interest in older adults with and without neurodegenerative disease (Nemy et al., 2020; Schumacher et al., 2022).

All regions of interest were identified using predetermined atlases in MNI space, which were then transformed to subject space by applying the nonlinear registration from T1-w to MNI to the diffusion image that was already aligned in the MNI ACPC plane. The internal capsule, external capsule, and cingulum were extracted from the Johns Hopkins University white matter atlas (Mori et al., 2005), the anterior commissure from FSL’s XTRACT atlas (Warrington et al., 2020), and the NBMs from the Ch4 basal forebrain regions identified by stereotaxic probabilistic maps from 10 high-quality post-mortem brains (Zaborszky et al., 2008). Consistent with the appropriate threshold for realistic NBM extraction (Wang et al., 2022), the probabilistic NBM masks were threshold at 50%.

### 2.6 Probabilistic tractography

After preprocessing the data with bedpostX, probabilistic tractography was completed using the FSL defaults of 5000 samples for each voxel within the seed mask and 0.2 curvature threshold settings in probtrackX. The NBM was used as the seed region, and the ipsilateral external capsule as the waypoint region of inclusion. The anterior commissure, internal capsule, brain stem, and hemisphere were specified as regions of avoidance. The output of probtrackX is a 3D streamline density image that contains the number of streamlines reaching each voxel. These streamlines originate from the seed region and pass through the waypoint but do not pass through any exclusion regions. This 3D image is then thresholded at 90% and binarized so that only the voxels with an intensity in the top 10% of the robust range remain. The decision of what level to threshold the tract outputs is always to some degree arbitrary. However, a 90% threshold appeared to be an effective threshold for minimizing the false positive connections while balancing the true positives. To determine whether higher thresholds would produce large discrepancies, we compared the dice coefficients of the tract outputs from run 1 cohort 1 when thresholding at 95%, 97%, or 99% to the 90% threshold.

To compare the similarity of the shape of the binarized tracts, Dice similarity coefficients were calculated using fsl_dice. Dice similarity coefficients are a metric of how identical two binary images are (Zou et al., 2004). A Dice coefficient equal to one is indicative of complete overlap while a score of zero indicates no overlap. Currently, there is no scoring system for what is considered a poor, average, or good dice score. However, it is expected that larger, easier to identify structures will produce higher dice scores (>0.9) while smaller, harder to identify structures may be in the range of 0.4-0.7 (Bazin et al., 2020; Laiton-Bonadiez et al., 2022). Given the very small size of the NBM and the high curvature of the lateral tract (which increases difficulty of probabilistic tract reconstruction), a Dice coefficient of ≥0.5 was considered good.

For comparisons whereby the tracts were extracted from two separate scans, a common-space was created using ANT Multivariate template construction using cross-correlation rigid-body registration and 10 iterations (Avants et al., 2011). The tracts and NBM masks from each run were then transformed into this space to allow for structural comparisons using Dice coefficients. In addition, visual inspection of the tracts was performed for each run of each subject to identify whether the tract was recreated successfully. A tract was considered to be successful if it resembled the expected anatomy of the NBM lateral tract (Mesulam & Geula, 1988).

### 2.7 Data analyses

For cohort 1, the spatial overlap of the tracts and NBM masks were compared using Dice coefficients between data processed separately for each pair of test-retest scans for the HCP dataset. A paired-samples t-test was used to investigate differences in test-retest reliability Dice coefficients between the manual segmentation (by KBW) and automated pipeline on the HCP data. A direct comparison of the tracts outputted by both pipelines was also compared across each run using Dice coefficients. However, as the comparison across pipelines may be more influenced by differences in tract length, heat maps were also created to visualize the overlap between pipeline outcomes. For cohort 2, which only included a single scan per participant, Dice coefficients were also used to investigate the intra- and inter-reliability of the manual segmentation pipeline on PD research scans. This was accomplished by running the pipeline two times per scan. Paired-samples t-tests were used to compare the reliability within-raters, and the tract overlap between-raters (RAC vs. KBW) for each run. For both cohorts 1 and 2, the tracts were pooled across hemispheres due to no known reason for there to be interhemispheric differences in tract reproducibility. Finally, for cohort 3, which also only included a single scan per participant, Dice coefficients were used to investigate the test-retest reliability of the manual segmentation (by RAC) approach for recreating the tracts of interest in clinical scans of people with PD. This was accomplished by running the pipeline two times per scan. An alpha of 0.05 was used to indicate statistical significance.

## 3 Results

The comparison between the 90% threshold and the 95%, 97%, and 99% thresholds for the reconstructed lateral NBM tract achieved a Dice coefficient of >0.94 for the manually segmented pipeline and >0.96 for the automated segmentation pipeline. This indicates a high level of consistency in the outcomes of lateral NBM tractography using thresholds of 90% and above, which was subsequently used for the rest of the analyses.

### 3.1 Cohort 1: manual vs. automated segmentation in healthy young adult research scans

**Fig. 2.**
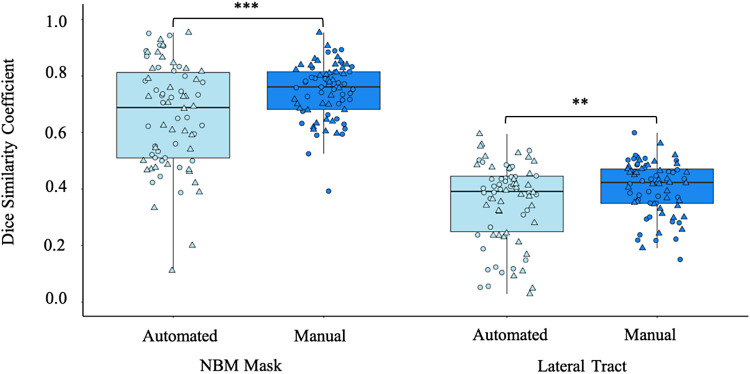
Comparison of the test-retest reliability of the automated and manual segmentation pipeline tractography outputs from healthy young adult high-quality research scans. Circle: Left hemisphere; Triangle: Right hemisphere. **p < 0.01, ***p < 0.001

Visual inspection of the data against post-mortem histological staining of the cholinergic fibres emanating from the NBM (Mesulam & Geula, 1988) identified a 97% success rate for run 1 and 99% success rate for run 2 in tract recreation for the manual segmentation approach compared to 86% for run 1 and 92% for run 2 of the automated segmentation pipeline. Two extreme outliers (Mean ± 3SD) were identified in the Dice coefficient of the automated NBM masks. However, removal of these data points did not alter the significance of the results and thus was kept in the final analyses. The individualized manual segmentation pipeline had significantly higher Dice coefficient than the existing automated pipeline across the HCP test-retest cohort for both the NBM mask itself (t = 4.24, p < 0.001) and the lateral NBM tracts (t = 3.17, p = 0.002). This indicates higher test-retest scan to scan reliability of the manual segmentation pipeline (see Fig. 2).

**Fig. 3.**
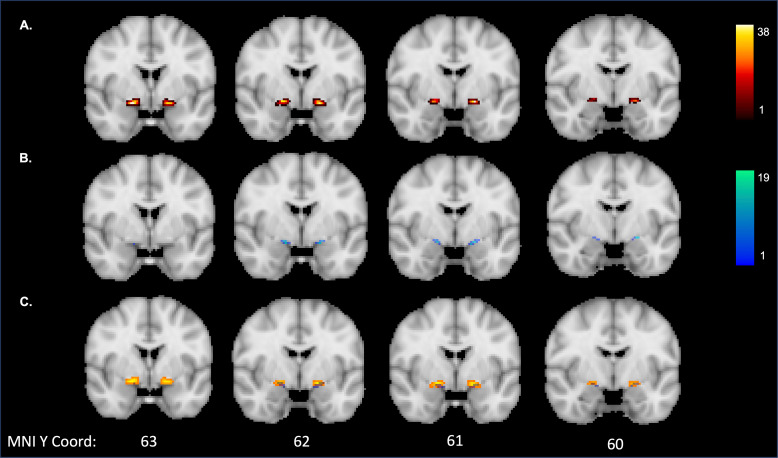
Overlap of the run 1 NBM masks in MNI space for: (A) manual segmentation pipeline; (B) automated segmentation pipeline; and (C) overlap between manual (orange) and automated pipeline (blue). The colour map indicates the number of participants with overlap at a given voxel.

In addition to test-retest reliability, we also sought to directly compare the outputs of each pipeline (i.e., manual vs. automated segmentations) across each run. The mean (SD) Dice coefficient between pipelines of the NBM mask itself was 0.16 (0.09) for run 1 and 0.15 (0.07) for run 2. The low Dice coefficients are most likely due to the much smaller volumes of the automated masks compared to the manually segmented masks (see Fig. 3).

**Fig. 4.**
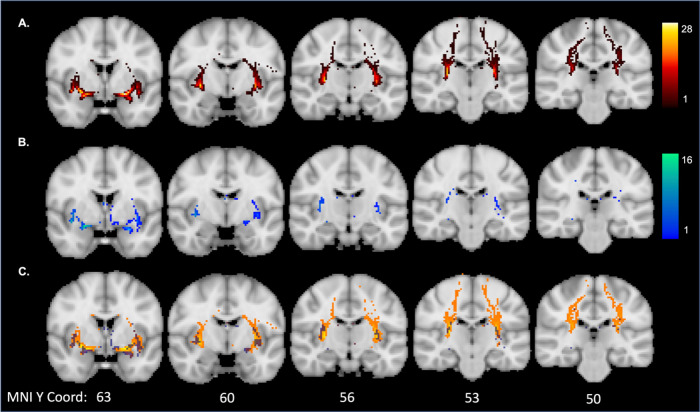
Overlap of the run 1 tract outputs in MNI space for: (A) manual segmentation pipeline; (B) automated segmentation pipeline; and (C) overlap between manual (orange) and automated pipelines (blue). The colour map indicates the number of participants with overlap at a given voxel. Mean (SD) volume was 19839 (917.0) for the manual segmentation tracts and 462.2 (279.2) for the automatic segmentation tracts.

For the tract outputs, the mean (SD) Dice coefficient between pipelines was 0.27 (0.17) for run 1 and 0.23 (0.15) for run 2. As the low Dice coefficient here may be due to a difference in the length of the tracts that were produced from each pipeline, we created heatmaps of the overlap between the tract outputs as well. Each tract was transformed to MNI space using the same registration step for registering the images to ACPC alignment described in Section 2.3. The tracts were binarized at threshold of 80% after being transformed to MNI space. Heatmaps reflecting the overlap of the tracts produced from each pipeline are displayed in Figure 4. Visual inspection of these results would suggest more overlap of the two pipelines at the tract segments directly connected to the seed. The manual segmentation pipeline had more overlap across participants of specific tract voxels than the automated segmentation pipeline. The latter pipeline was also much shorter than the manually segmented pipeline, suggesting more of the tract was successfully recreated in the manual pipeline.

### 3.2 Cohort 2: inter- and intra-rater reliability of the manual segmentation pipeline in patients with PD

**Fig. 5.**
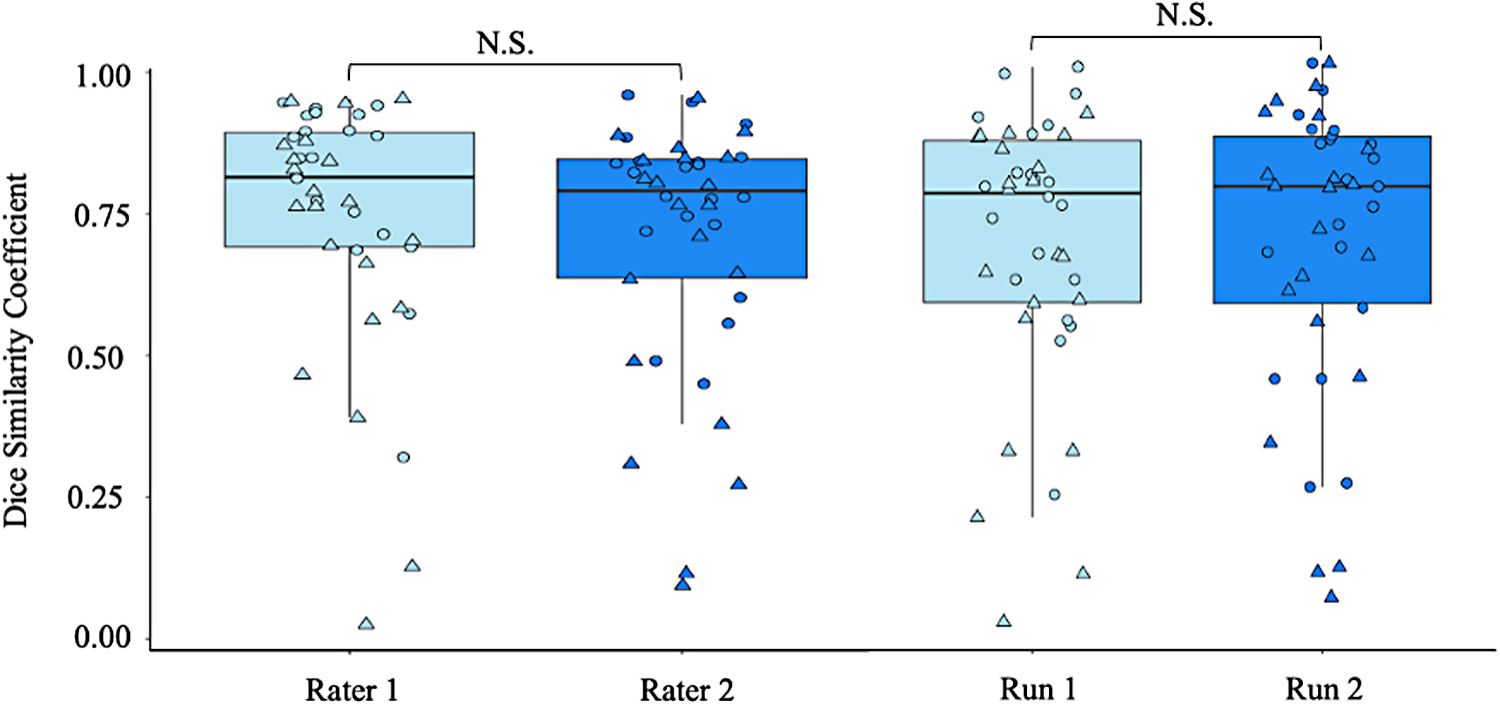
Left: comparison of the test-retest reliability for two different raters; and Right: comparison of the inter-rater reliability across each run using data from the Parkinson’s Progression Markers Initiative. Circle: Left hemisphere; Triangle: Right hemisphere; N.S. Not significant.

When running the pipeline on research quality scans in PD patients who may be eligible for NBM DBS, visual inspection of the tractography outputs identified an 83% and 79% success rate for rater 1 across run 1 and 2 respectively and a 79% and 71% success rate for rater 2. We found high Dice coefficients for both raters between runs for the NBM lateral tract (mean >0.70) indicating high intra-rater reliability. Notably, there was no significant difference (t = 1.17, p = 0.25) in the intra-rater Dice coefficient between raters, suggesting both raters were equally reliable. In addition, there was no significant difference in the inter-rater reliability for each run (t = -0.31, p = 0.75). The mean between-rater Dice coefficient was 0.65 for run 1 and 0.66 for run 2, indicating high inter-rater reliability of the pipeline (see Fig. 5).

### 3.3 Cohort 3: reliability of manual segmentation pipeline in clinical scans

**Fig. 6.**
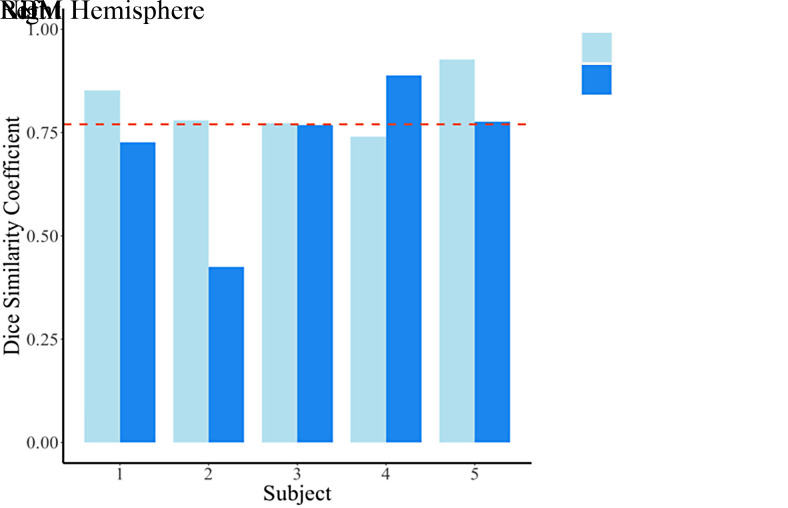
Dice similarity coefficients for the test-retest reliability of the manual segmentation tractography approach in clinical scans. The dashed line indicates the mean Dice coefficient (0.77) across the full sample.

The mean (SD) Dice coefficient for the clinical scans was 0.77 (0.1), which is indicative of very high reliability across runs (see Fig. 6). Visual inspection of the data demonstrated a 70% success rate for both runs in the reconstruction of the tract of interest. The target tracts were not successfully recreated across either run for both hemispheres in subject 2 and for the right side in subject 1.

## 4 Discussion

We successfully developed a novel individualized tractography pipeline for the reconstruction of the NBM lateral tracts. The pipeline was found to have greater test-retest reliability than existing automated segmentation methods in high-quality MRI scans of healthy young adults. We also found high inter- and intra-rater reliability across research and clinical grade MRI scans in patients with PD who are eligible for DBS. However, greater consideration may be needed when using this approach in clinical neuroimaging of patients with high levels of atrophy and lower quality scanning parameters.

Previous methods to identify and recreate the NBM tracts have used automated NBM segmentation methods. However, these automated segmentation methods are based on masks created from small samples of post-mortem brains and have been shown to produce inconsistent seed volumes (Wang et al., 2022), which may lead to variability in subsequent identified tracts. More recently, Doss et al. (2023) used deep learning methods to improve the accuracy of automated segmentation approaches. However, this approach still relied on the initial manual identification of the NBM in a healthy cohort, which was then translated to a clinical population of temporal lobe epilepsy patients. We expanded on the manual segmentation work by George et al. (2011) by narrowing the anatomical focus to that of the NBM and using both structural and diffusion-weighted imaging for enhanced visualization of the region of interest. It is notable that the outcome of the tractography produced from manually identifying the NBM was more successful in recreating the lateral NBM tract when combined with the manual identification of the anterior commissure rather than using existing automated masks which struggled to identify the full anatomy of this region. The anterior commissure is also used as a region of exclusion within the probabilistic tractography pipeline. Therefore, the manual segmentation of this region has the additional benefit of providing greater guidance for the NBM tract reconstruction.

Overall, we identified that our manually segmented individualized pipeline recreated more consistent NBM masks and subsequently the NBM lateral tracts than using automated mask segmentations, indicating improved test-retest reliability. It is notable that the overall inter-scan Dice coefficient for the HCP cohort was lower than when assessing the reliability of the pipeline in the same scan for the PPMI and DBS patient cohorts. Consequently, the reliability of both approaches is slightly reduced when inter-scanner/repeat scanning is a factor. Of note, instances of successful outcomes were evident by the expected connections from the NBM, through the external capsule and fanning into the cortex. However, in the few unsuccessful outcomes, it appeared the tracts mostly resembled the optic radiation. While the manual segmentation pipeline did have greater success than the automated segmentation, outliers were evident in the lower quality scans of PD patients (cohorts 2 and 3) and were consistent across raters. This was likely due to the differences in scanning parameters. However, disease progression may also have contributed.

While the manual segmentation approach may lead to more precise tractography outcomes, it can also be vulnerable to greater inter-rater variability. Thus, we sought to evaluate the reliability of this pipeline across two trained researchers. We identified high inter- and intra-rater reliability between researchers and across tractography runs respectively. Importantly, there was no significant difference between the dice coefficients comparing each rater across two runs of tractography. This indicates that both raters were equally reliable at recreating the tracts of interest. In addition, this analysis was shown to be reliable within a cohort of PD patients who would be eligible for NBM DBS (i.e., had mild cognitive impairment), therefore indicating the benefit of this approach for both healthy young adults and those with neurodegenerative disease.

Finally, we aimed to investigate the use of this tractography approach for clinical use. We identified high test-retest reliability in the clinical scans. However, there were three tracts which were not reproducible for either run. This is likely a result of lower quality imaging parameters. For instance, compared to the 1.25 mm^3^ voxel size, 90 gradient directions, and three *b* values of the high-quality scans from the HCP, clinical MRI generally only use a 2 mm^3^ voxel size, 30 gradient directions, and only one *b* value. This reduces the sensitivity of the scan to detect accurate fiber orientations. The smaller voxel size is also particularly relevant for spatial resolution given the very small size of the NBM (~3 x 13.5 x 17 mm) (Jethwa et al., 2019; Mesulam & Geula, 1988). Thus, the larger slice thickness in clinical scans may lead to either over or under estimation of the NBM region itself.

The need to develop improved research tools to examine the integrity of these major white matter tracts is of vital importance. A growing body of evidence suggests changes in white matter may be more sensitive at identifying differences in clinical populations than grey matter. Taylor et al. (2018) showed that changes in white but not grey matter integrity were associated with changes in symptom severity in de-novo PD patients over 12 months. This supports the finding that deficits to the lateral NBM white matter fiber bundle, but not the grey matter nuclei itself, were predictive of cognitive decline in patients with Alzheimer’s disease or dementia with Lewy bodies as well (Schumacher et al., 2022). We have also highlighted the reduced integrity of the NBM white matter tracts up to one year prior to the development of cognitive impairment in untreated, early-stage PD patients (Crockett et al., 2023). In addition to neurodegenerative disease, reduced integrity of the white matter was identified in patients with Euthymic bipolar I disorder compared to controls (Emsell et al., 2013). However, no differences were identified between groups in the grey matter. These findings suggest a greater need for neuroimaging analysis tools that can unlock new white matter targets of investigation. The ability to expand the focus of future research will provide vital insight into the underlying neurobiology of a multitude of neurological diseases, which is crucial for early detection and treatment.

This study is not without limitations. Firstly, the lower Dice coefficient values across both pipelines in the HCP data suggest an impact of inter-scanner noise on the consistency of tractography outputs. We did not have repeat scans at the same timepoint for the PPMI and clinical data, which reduced our ability to investigate the test-retest scan to scan reliability across different scanning procedures in the PD population. We also chose to only assess the reliability of these pipelines using the FSL probabilistic method of tractography. While this is considered the most robust method (Lilja et al., 2014; Zhan et al., 2015), we did not assess how these approaches may compare across other tractography algorithms, such as deterministic tractography, that may also be used in research and/or clinical settings. Lastly, at present, there is no ground truth for the identification of the lateral NBM tract, which would be needed for determining the sensitivity and specificity of the tractography pipelines. Therefore, the interpretation of these tract outputs is limited to visual comparison with histological maps of target tract. However, the nature of these tracts from these histological studies is well documented (Mesulam & Geula, 1988; Selden, 1998).

## 5 Conclusion

Our novel individualized tractography pipeline was shown to be highly reliable at reconstructing the lateral NBM tracts in both healthy young adults and patients with PD. This pipeline can be used for improved reliability of research investigating the cortical cholinergic network in aging and neurodegenerative populations and can inform the neurosurgical planning of DBS aimed at targeting this network.

## Supplementary Material

Supplementary Material

## Data Availability

Data from the Human Connectome Project can be accessed upon request at https://www.humanconnectome.org/study/hcp-young-adult/data-releases, and data from the Parkinson’s Progression Markers Initiative (PPMI) database can be accessed upon request at www.ppmi-info.org/access-data-specimens/download-data. For up-to-date information on the PPMI study, visit www.ppmi-info.org.
