# Supplementary material for: An individualized tractography pipeline for the nucleus basalis of Meynert lateral tract
Source: Imaging Neurosci (Camb). 2024 Jan 11;2:imag-2-00067. doi: 10.1162/imag_a_00067 (PMC12148219; doi:10.1162/imag_a_00067)
Supplement: Supplementary Material [file imag_a_00067-supp.pdf]

Supplementary Material (S1)

A guide for manually segmenting the NBM using neighbouring anatomy slice-by-slice in the MNI space. An explanation of the layout for each slice is provided in Figure S1.

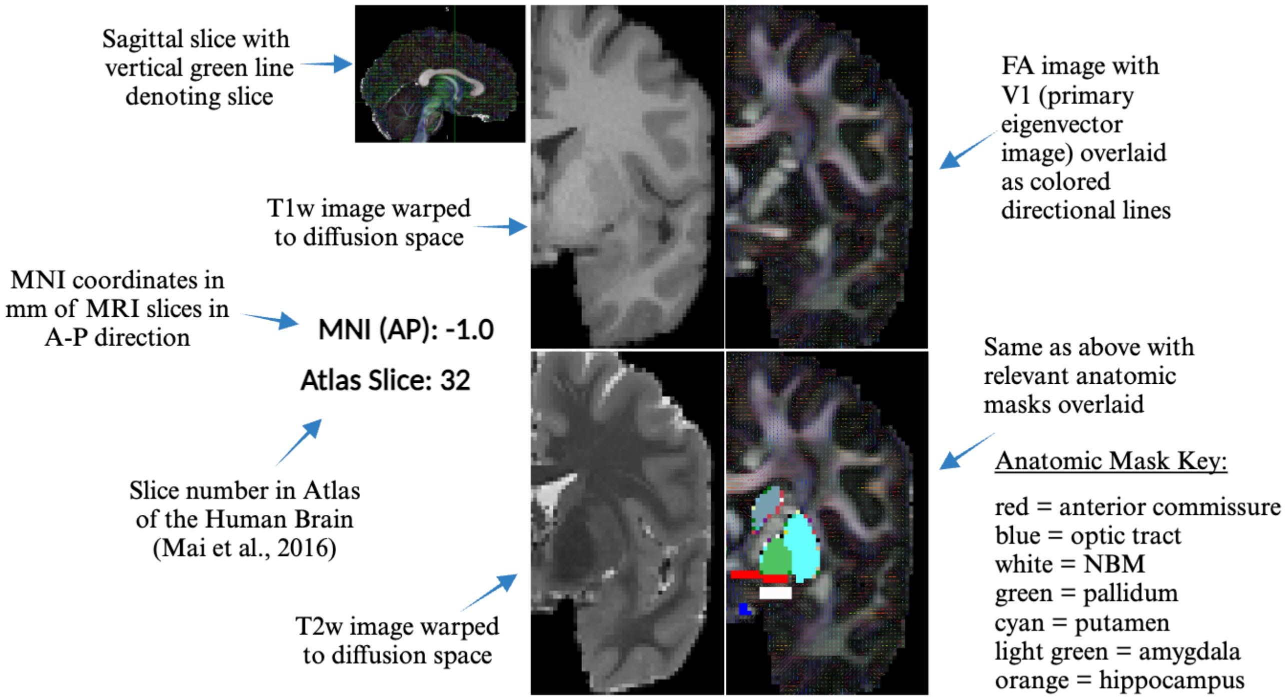

Figure S1. An overview of the layout for the slice-by-slice example of NBM segmentation.

Visual Set-up in FSLeyes

Within FSLeyes, add the FA and V1 images from the DTIFIT output. Set the overlay of the V1 image to be a 3-direction vector image to create the directional line overlay. Then add the landmark region masks: optic tract, anterior commissure, and all_fast_first_seg output from the FIRST segmentation. Set the color scheme for the FIRST output to be MGH sub-cortical. For the NBM segmentation, only the coronal view is needed.

Overview of Landmarks

- *Anterior Landmark:* At least one slice posterior to the anterior commissure. In scans where identification of the anterior commissure is challenging, the point at which the inferior end of the fornix column meets the base of the internal capsule can be used as an additional indicator of the anterior NBM boundary.
- *Posterior Landmark:* The point whereby the hippocampus begins to become evident, and the optic tract starts to slide laterally underneath the pallidum.
- *Dorsal Landmark:* Pallidum.
- *Ventral Landmark:* Amygdala.
- *Medial Landmark:* Aligned with the medial edge of the pallidum.
- *Lateral Landmark:* Aligned with the border between the lateral edge of the pallidum.
- *Additional avoidance:* While segmenting within these landmarks it is important to avoid overlapping with any portion of the anterior commissure and optic tract.

Full example of NBM segmentation by MNI slice

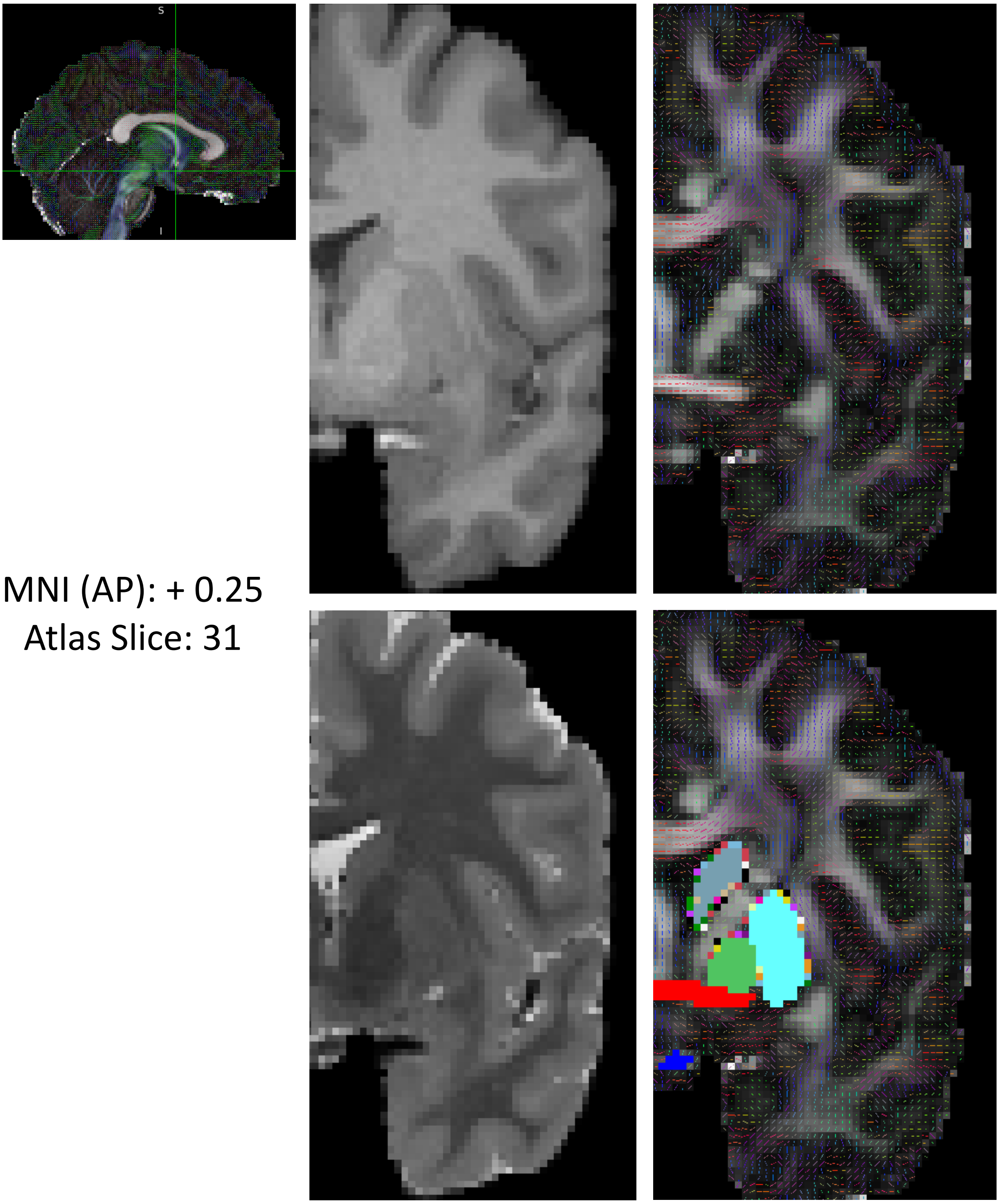

MNI (AP): + 0.25
Atlas Slice: 31

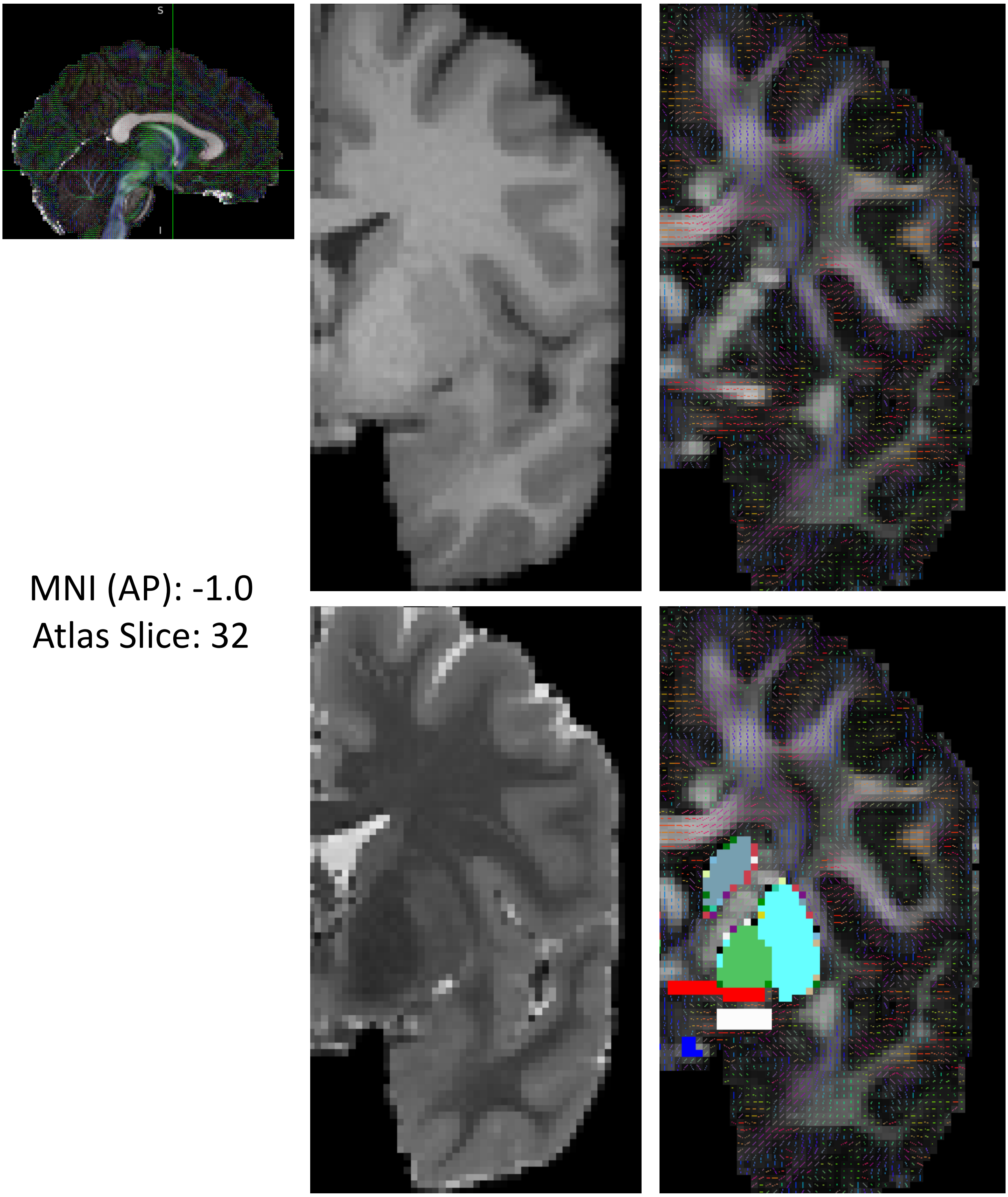
MNI (AP): -1.0
Atlas Slice: 32

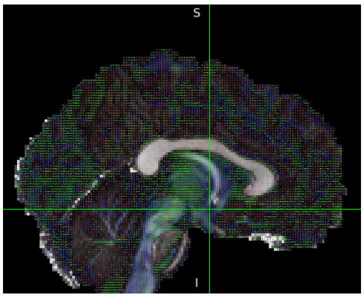

MNI (AP): -2.25
Atlas Slice: 34

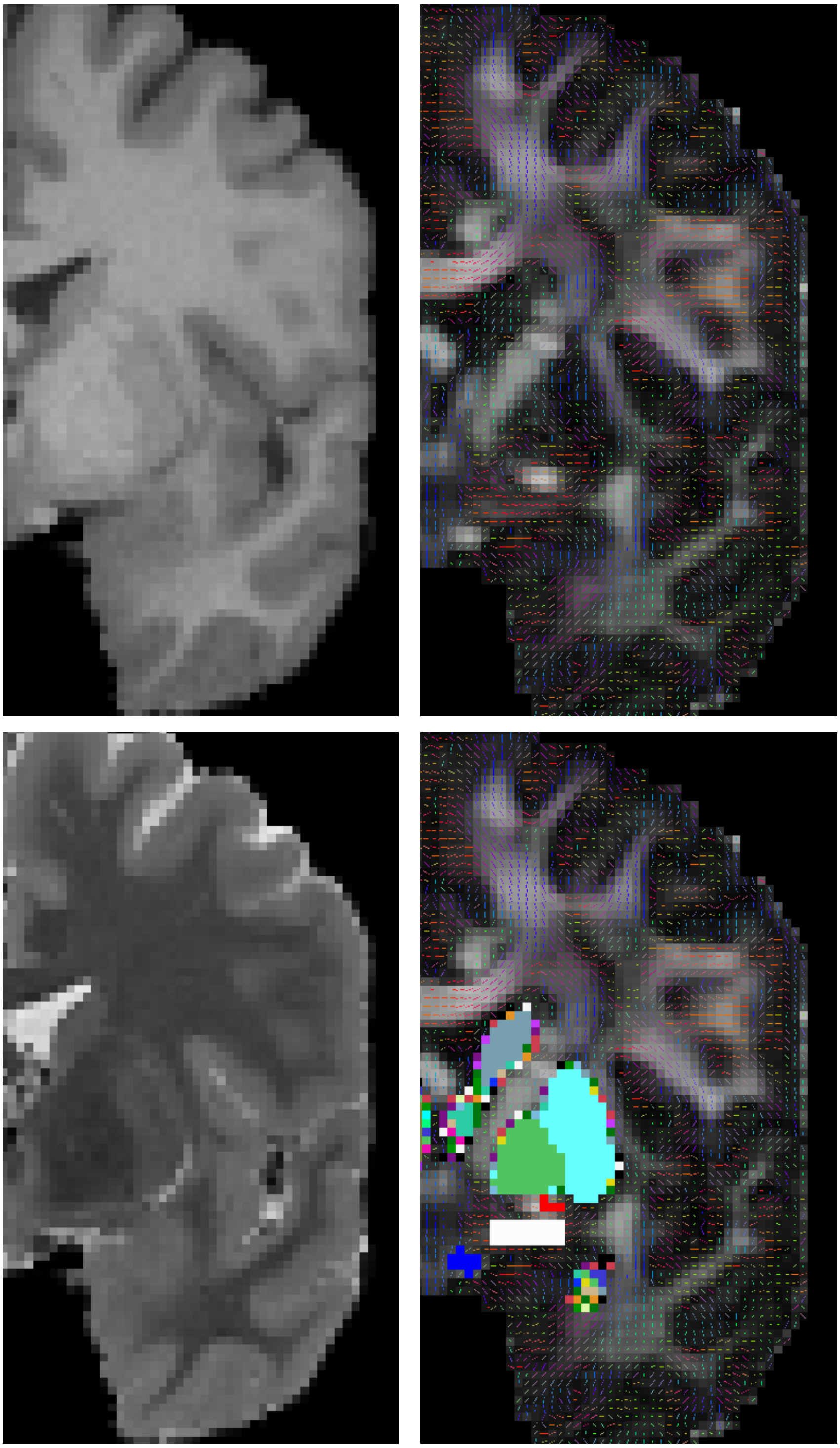

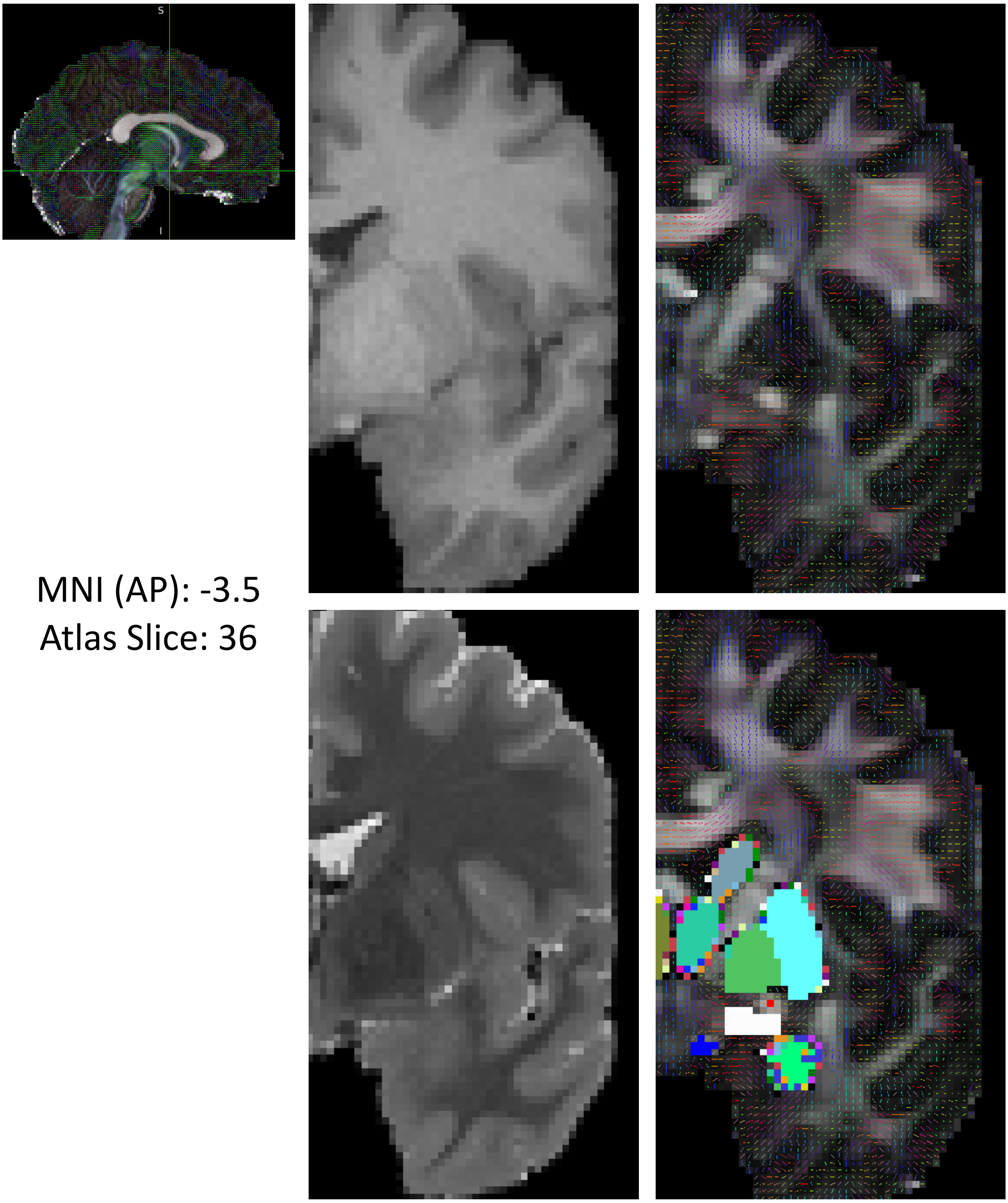

MNI (AP): -3.5
Atlas Slice: 36

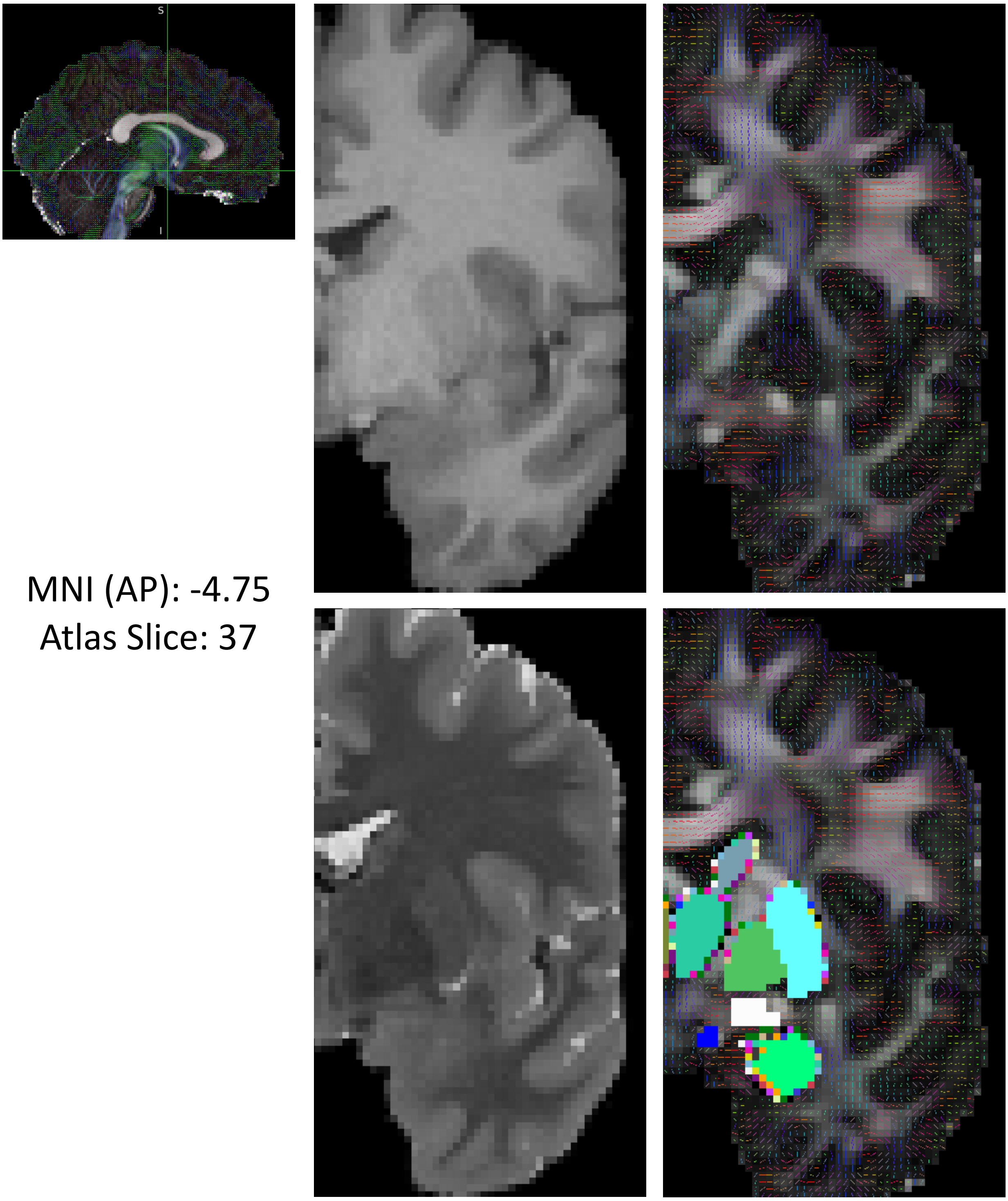

MNI (AP): -4.75
Atlas Slice: 37

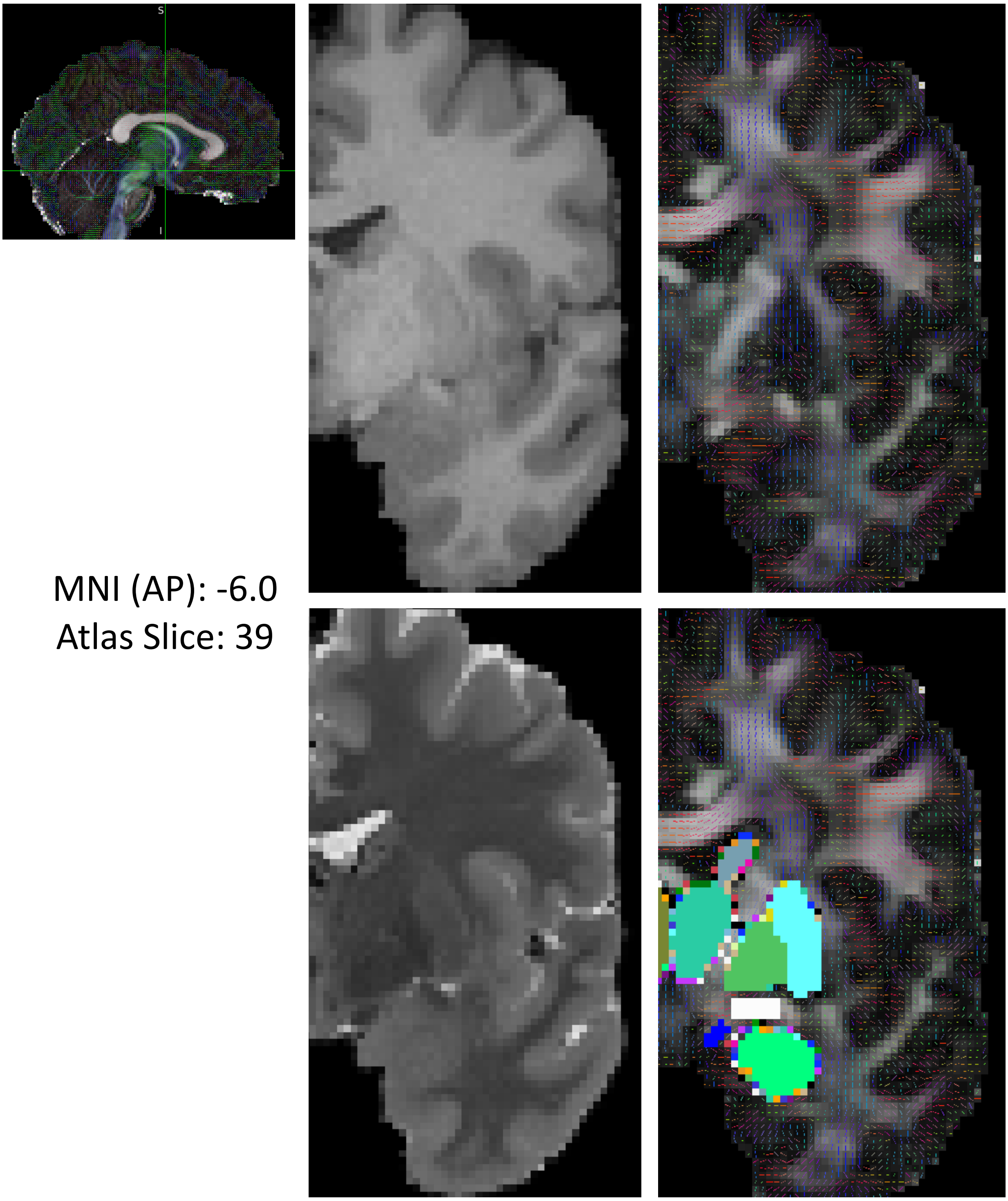
MNI (AP): -6.0
Atlas Slice: 39

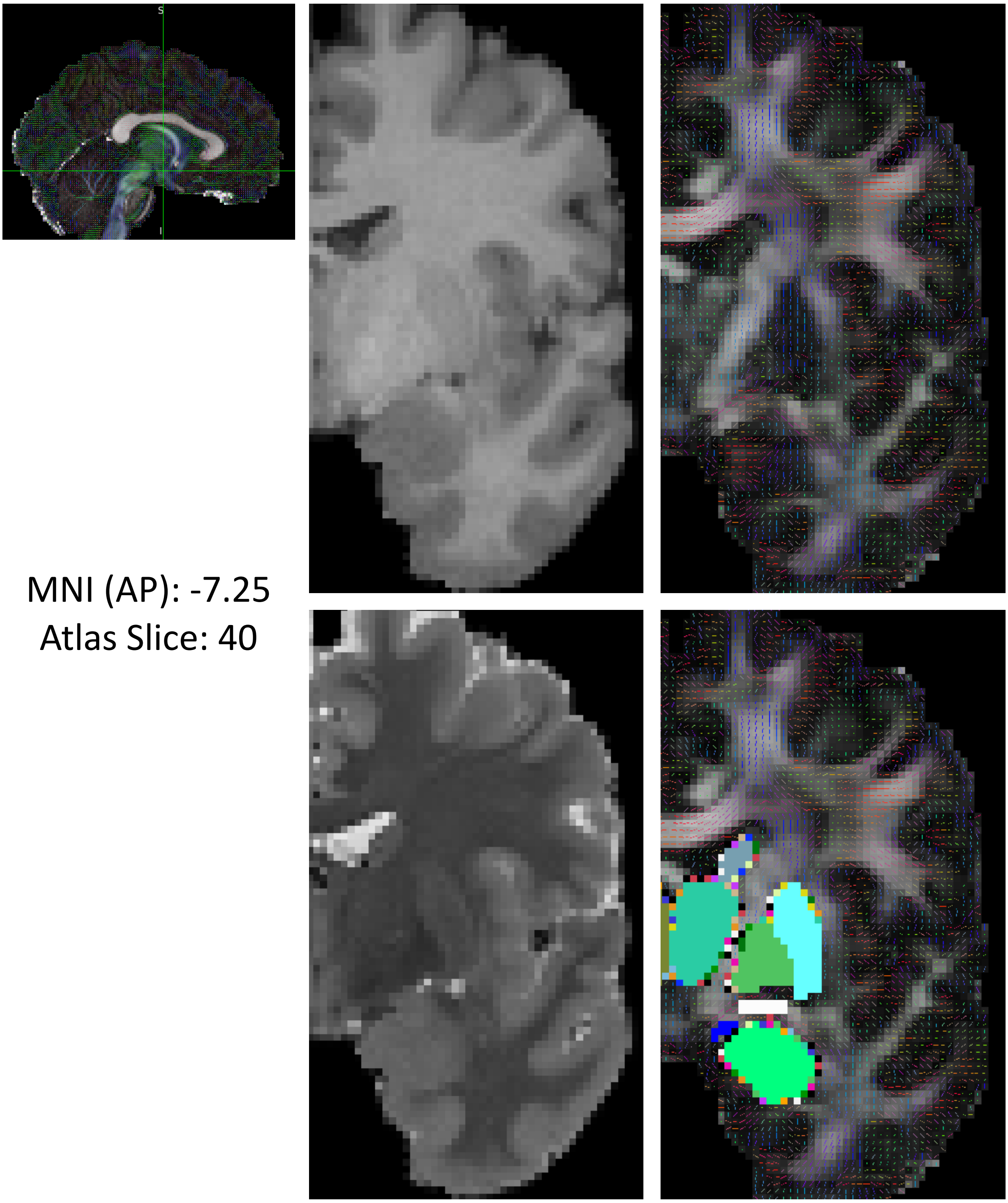

MNI (AP): -7.25
Atlas Slice: 40

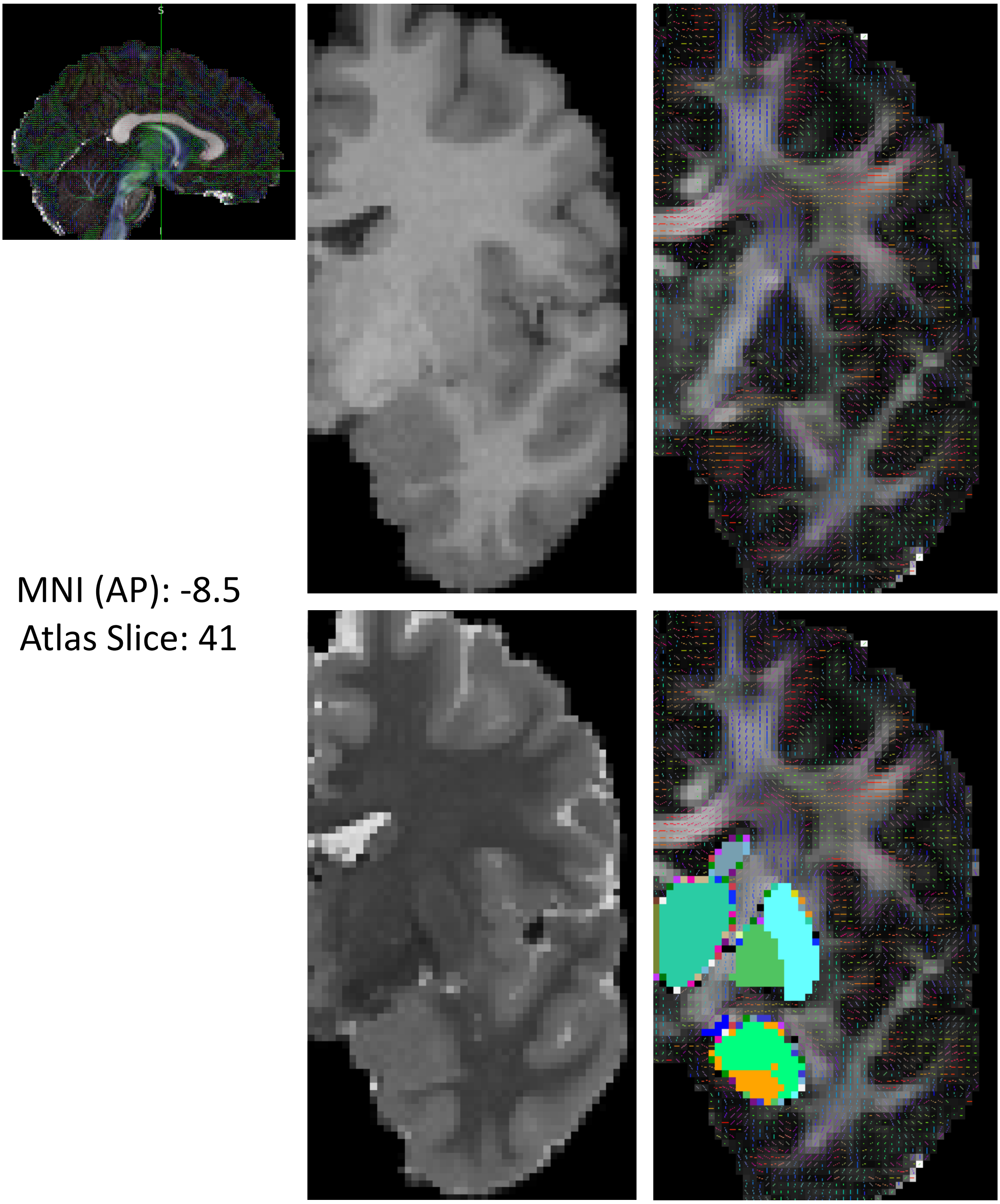

MNI (AP): -8.5
Atlas Slice: 41

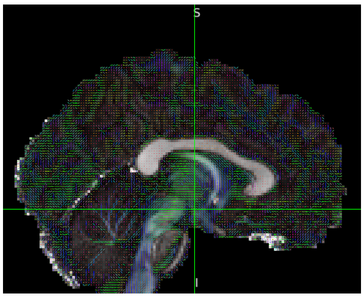

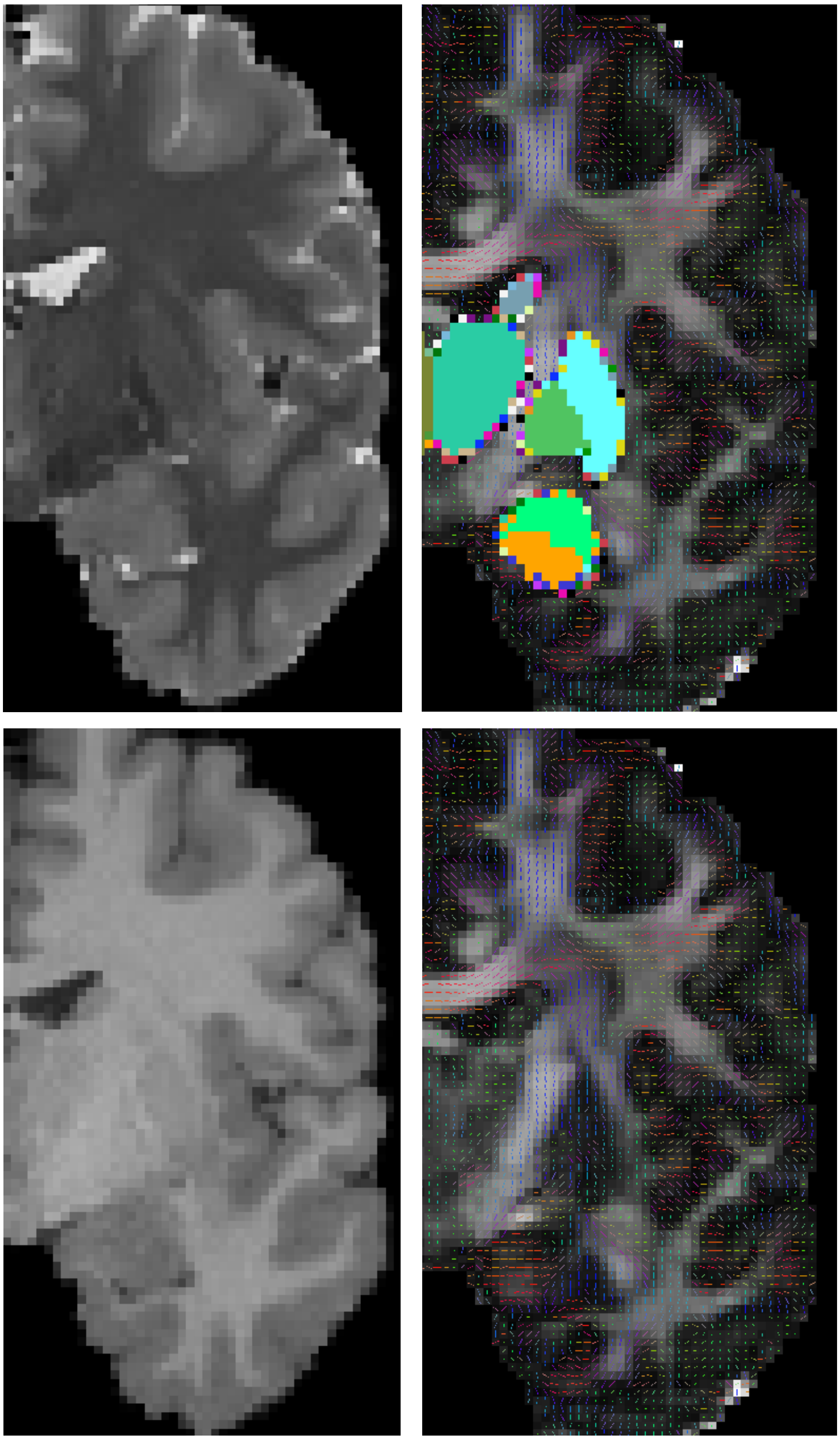

MNI (AP): -9.75
Atlas Slice: 42
